# Analysis of the effectiveness of interventions used during the 2009 A/H1N1 influenza pandemic

**DOI:** 10.1186/1471-2458-10-168

**Published:** 2010-03-29

**Authors:** Nilimesh Halder, Joel K Kelso, George J Milne

**Affiliations:** 1 School of Computer Science and Software Engineering, University of Western Australia, Perth, Australia

## Abstract

**Background:**

Following the emergence of the A/H1N1 2009 influenza pandemic, public health interventions were activated to lessen its potential impact. Computer modelling and simulation can be used to determine the potential effectiveness of the social distancing and antiviral drug therapy interventions that were used at the early stages of the pandemic, providing guidance to public health policy makers as to intervention strategies in future pandemics involving a highly pathogenic influenza strain.

**Methods:**

An individual-based model of a real community with a population of approximately 30,000 was used to determine the impact of alternative interventions strategies, including those used in the initial stages of the 2009 pandemic. Different interventions, namely school closure and antiviral strategies, were simulated in isolation and in combination to form different plausible scenarios. We simulated epidemics with reproduction numbers R_0_of 1.5, which aligns with estimates in the range 1.4-1.6 determined from the initial outbreak in Mexico.

**Results:**

School closure of 1 week was determined to have minimal effect on reducing overall illness attack rate. Antiviral drug treatment of 50% of symptomatic cases reduced the attack rate by 6.5%, from an unmitigated rate of 32.5% to 26%. Treatment of diagnosed individuals combined with additional household prophylaxis reduced the final attack rate to 19%. Further extension of prophylaxis to close contacts (in schools and workplaces) further reduced the overall attack rate to 13% and reduced the peak daily illness rate from 120 to 22 per 10,000 individuals. We determined the size of antiviral stockpile required; the ratio of the required number of antiviral courses to population was 13% for the treatment-only strategy, 25% for treatment and household prophylaxis and 40% for treatment, household and extended prophylaxis. Additional simulations suggest that coupling school closure with the antiviral strategies further reduces epidemic impact.

**Conclusions:**

These results suggest that the aggressive use of antiviral drugs together with extended school closure may substantially slow the rate of influenza epidemic development. These strategies are more rigorous than those actually used during the early stages of the relatively mild 2009 pandemic, and are appropriate for future pandemics that have high morbidity and mortality rates.

## Background

A novel strain of the A/H1N1 influenza virus has rapidly spread around the world, leading to the first influenza pandemic since 1968. While current data indicates that this influenza virus results in mild symptoms and relatively low mortality characteristics, it allows us to examine interventions strategies that will be necessary for a future highly pathogenic influenza strain when, as with the 2009 pandemic, no suitable vaccine will be initially available. The 2009 pandemic virus first appeared in Mexico in April 2009 and later spread around the world, causing at least 15292 deaths as of 12th February 2010 [1]. Due to its worldwide spread the World Health Organization (WHO) lifted its pandemic alert to phase 6, the highest alert phase. Phase 6 indicates human-to-human transmission of a novel influenza strain with sustained community level outbreaks in two or more countries in one WHO region and community level outbreaks in at least one other country in another region. As of the 12^th ^February 2010; 37,693 laboratory confirmed cases and 191 deaths have been reported in Australia [2].

The use of both pharmaceutical and social distancing interventions are embedded within the pandemic preparedness plans of most countries [3-5] and also appear in recent WHO recommendations [6]. School closure, household quarantine and reduced workplace, social and community contacts are considered to be key non-pharmaceutical interventions which may readily be used for the early containment of an influenza epidemic. These social distancing measures have been shown in modelling studies to delay the overall impact of a pandemic, as well as allowing time for antiviral drug administration and the development of appropriate vaccines [7,8]. Neuraminidase inhibitor antiviral drugs and appropriate vaccines are the key pharmaceutical interventions, with antiviral drugs being the only available pharmaceutical interventions available when faced with a novel strain of influenza virus in the early phase of pandemic, as has occurred in 2009. Social distancing measures, such as school closure, and antiviral drug strategies have been used in the initial stages of the A/H1N1 2009 pandemic in Australia and other parts of the world [9,10].

The effectiveness of the various pandemic containment measures used is not fully understood due to a lack of field data [11]. Simulation models have therefore been developed to understand the dynamics of pandemics and to analyze the benefit of potential containment strategies, such as those that are recommended in pandemic preparedness plans. Modelling techniques for the study of infectious respiratory diseases such as pandemic influenza have included deterministic [12-14], stochastic [15-17] and individual-based models [18-25]. These studies use models that range in scale from the whole world [12,15,16], through large [20,22] and small [19,21] countries, to actual [7,8] and synthetic [23,26] small communities. The outcome of modelling studies used to assess the effectiveness of various proposed interventions are sensitive to the operational details of the interventions (such as the timing and duration of school closure [7,27,28]) and to the particular combination of interventions used. Prior to the occurrence of a pandemic, modelling studies have made plausible assumptions about these details. However, due to the number of combinations of intervention types, variation in intervention details and possible emergent virus characteristics, exhaustive analysis has been impossible. Having now observed the response to a pandemic, we are in a position to model in detail several different intervention strategies which were actually used and which, therefore, are highly likely to be considered for use in future pandemics or in resurgent waves of the current pandemic. Furthermore, we have focussed on epidemics with the estimated characteristics of the A/H1N1 2009 pandemic.

The purpose of this study was to examine the interventions used in Australia (and subsequently in other countries) during the early stages of the 2009 influenza pandemic, to determine their effectiveness (in reducing the illness attack rate) and cost (in terms of the required number of antiviral courses), and further determine whether alternative intervention strategies would have been more effective. We modelled the spread dynamics within a simulation model using virus characteristics estimated from data obtained at an early phase of the Mexico epidemic [29], and we simulated the key features of the actual school closure and antiviral-based strategies used in the initial stages of the outbreak in Australia. We present results from an examination of a range of school closure and antiviral drug strategies, singly and in combination, to determine their effectiveness. The results suggest strategies that are more effective than those recently used (in Australia and other countries) and which may need to be considered by public health authorities as they revise their Pandemic Preparedness Plans. The results provide guidance as to the optimum strategies required to contain or significantly limit the impact of a future, possibly highly pathogenic, pandemic influenza strain.

## Methods

We used a detailed individual-based model of a real community in the south west of Western Australia (Albany) with a population of approximately 30,000 to simulate the dynamics of the 2009 influenza pandemic [7,8,30].

### Population contact network

The simulation model captures the contact dynamics of the population of Albany, Western Australia using census and state and local government data [31], allowing us to replicate the individual age and household structure of all households in this town of approximately 30,000 individuals. Human contact networks were modelled as a network of connected households and contact hubs such as schools, childcare centres, workplaces and a regional hospital. Individuals in each household and hub made contacts within a close contact mixing group, taken to be the entire household or a subset of larger hubs, and also made additional non-hub based random contacts in the wider community. Using this community-based population model, we conducted stochastic, individual-based spatial simulations of the A/H1N1 2009 swine flu strain currently circulating in Australia. We assumed that an average of one new infection per day was stochastically introduced into the population during the whole period of the simulations. The simulation period was divided into 12 hour day/night cycles and during each simulation cycle a nominal location of each individual was determined; taking into consideration the cycle type (day/night, weekday/weekend), infection state of each individual and whether child supervision was needed to look after a child at home. Individuals occupying the same location during the same time period (cycle) were assumed to come into potential infective contact. Details of the underlying model are presented in [7] and in that reference's online supporting material.

### Influenza transmission model

**Figure 1:**
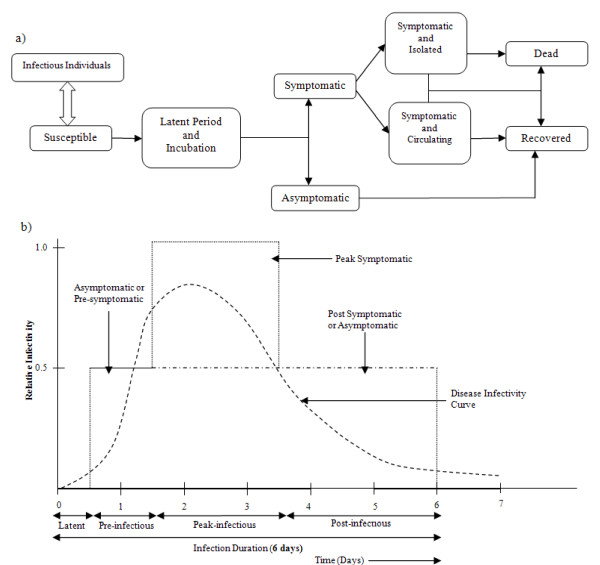
**Dynamics of influenza progression within host individuals**. (a) Dynamics of influenza A/H1N12009 progression within host individuals. (b) Infectivity curve of an infectious individual due to influenza infection.

In the simulation model we assumed that infectious transmission could occur when an infectious and susceptible individual came into contact during a simulation cycle. Following each contact a new infection state for the susceptible individual (either to remain susceptible or to become infected) was randomly chosen via a Bernoulli trail [32]. Once "infected" an individual progressed through a series of infection states according to a fixed timeline. The SEIR state progression dynamics of individuals is illustrated in Figure 1a.

The transmission probability that a susceptible individual would be infected by an infectious individual was calculated according to the following transmission function, which takes into account the disease infectivity of the infectious individual ***I*_*i *_**and the susceptibility of susceptible individual ***I*_*s *_**at the time of contact. A description of each component of the following probability function is given in Table 1:

**Table 1:** Detailed description of each component of the transmission function

Component of transmission function	Description
**P_*trans*_(*I*_*i*_, *I*_*s*_)**	Probability of disease transmission from *infected *to *susceptible *individual.
***β***	Disease transmission coefficient, chosen to achieve baseline epidemics with specific R_0 _values.
**Susc(*I*_*s*_)**	Age specific susceptibility of *infectee *or *susceptible *individual.
**Inf_t_(*I*_*i*_)**	Infectiousness of *infector *as a function of time since infection and symptomatic status.
**AVF(*I*_*i*_, *I*_*s*_)**	Antiviral factor which reduces transmission probability when antiviral based interventions are applied to individuals ***I*_*i*_, *I*_*s *_**or both.
The following different situations may arise when we model antiviral effectiveness.	
**AVF(*I*_*i*_, *I*_*s*_) = 1 - AVE_*i*_**	if the infected individual is treated with antiviral drugs and no antiviral prophylaxis is applied to the susceptible individual
**AVF(*I*_*i*_, *I*_*s*_) = 1 - AVE_*s*_**	if antiviral prophylaxis are applied to the susceptible individual and no antiviral treatment is used with the infected individual
AVF(*I*_*i*_, *I*_*s*_) = (1-AVE_*i*_)*(1-AVE_*s*_)	if both infected and susceptible individuals are receiving antiviral drugs for treatment and prophylaxis respectively
**AVF(*I*_*i*_, *I*_*s*_) = 1**	if neither infector nor susceptible individuals are receiving antiviral drugs

The baseline transmission coefficient ***β ***was initially chosen to give an epidemic with a final attack rate of 17.4% which is consistent with seasonal influenza as estimated in Table three of [33]. To achieve simulations under a range of reproductive numbers, ***β ***was increased from this baseline value to achieve epidemics of various R_0 _magnitudes; Details of the procedure for estimating ***β ***and R_0 _are given in [7]. The basic reproductive number R_0 _for A/H1N1 2009 influenza virus was initially estimated to be between 1.4 and 1.6 [29,11]; more recent estimates range from 1.2 [34] to 2.3 [9]; We assume an R_0 _of 1.5 as being the midpoint of both ranges.

The disease infectivity parameter **Inf(*I*_*i*_) **was set to 1 for symptomatic individuals at the peak period of infection and then to 0.5 for the rest of the infectivity period The infectiousness of asymptomatic individuals is also assumed to be 0.5 and this applies to all infected individuals after the latent period but before onset of symptoms (see Figure 1b). The infection profile of a *symptomatic *individual was assumed to last for 6 days as follows: a 0.5 day latent period (with **Inf(*I*_*i*_) **set to 0) is followed by 1 day asymptomatic and infectious, where **Inf(*I*_*i*_) **is set to 0.5; then 2 days at peak infectiousness (with **Inf(*I*_*i*_) **set to 1.0); followed by 2.5 days reduced infectiousness (with **Inf(*I*_*i*_) **set to 0.5). For an infected but *asymptomatic *individual the whole infectious period (of 5.5 days) is at the reduced level of infectiousness with **Inf(*I*_*i*_) **set to 0.5. This infectivity profile is a simplification of the infectivity distribution found in a study of viral shedding [35]. As reported below in the results section for the unmitigated no intervention scenario, these assumptions regarding the duration of latent and infectious periods lead to a mean generation time (serial interval) of 2.47 days which is consistent with that estimated for A/H1N1 2009 influenza [29].

Following infection an individual is assumed to be immune to re-infection for the duration of the simulation. We further assume that influenza symptoms develop one day into the infectious period [35], with 20% of infections being asymptomatic among children and 32% being asymptomatic among adults. These percentages were derived by summing the age-specific antibody titres determined in Table five of [36]. Symptomatic individuals will withdraw into the home with the following probabilities; adults 50% and children 90%, which is in keeping with the work of [20,21].

The susceptibility parameter **Susc(*I*_*s*_) **is a function directly dependent on the age of the susceptible individual. It captures age-varying susceptibility to transmission due to either partial prior immunity or age-related differences in contact behaviour. To achieve a realistic age specific infection rate, the age-specific susceptibility parameters were calibrated against the serologic infection rates for seasonal H3N2 in 1977-1978 in Tecumseh, Michigan [33]. The resulting age-specific attack rates are consistent with A/H1N1 2009 influenza [37], with a higher attack rate in children and young adults (details may be found in [7]).

The antiviral efficacy factor AVF(*I*_*i*_, *I*_*s*_) = (1 - AVE_*i*_)*(1 - AVE_*s*_) represents the potential reduction in infectiousness of an infected individual (denoted by **AVE**_*i*_) induced by antiviral treatment, and the reduction in susceptibility of a susceptible individual (denoted by **AVE_*s*_**) induced by antiviral prophylaxis. When no antiviral intervention was administrated the values of both **AVE_*i *_**and **AVE_*s *_**were assumed to be 0, indicating no reduction in infectiousness or susceptibility. However, when antiviral treatment was being applied to the infectious individual the value of **AVE_*i *_**was set at 0.66, capturing a reduction in infectiousness by factor of 66% [38]. Similarly, when the susceptible individual was undergoing antiviral prophylaxis the value of **AVE_*s *_**was set to 0.85 indicating a reduction in susceptibility by a factor of 85% [38]. This estimate is higher than most previous modelling studies, which assume an AVE of 30% (e.g. [20,22,27]). This common assumption appears to stem from an estimate made in [26] based on 1998-1999 trial data. Our higher value is based on a more comprehensive estimation process reported in [38], which also incorporated an data from an additional study performed in 2000-2001 [39]. It is also in line with estimates of 64%-89% reported in [40].

### School closure and antiviral drug interventions

We analysed three different school closure strategies and three different antiviral intervention strategies that were used in Australia, the United Kingdom and the USA during the early stages of the 2009 influenza pandemic. The mitigating effect attained when applied to an outbreak of influenza A/H1N1 swine flu within the simulated Albany community was determined by comparing the resulting daily and cumulative illness attack rates with that of an unmitigated outbreak. We assumed that an infected, symptomatic individual would be diagnosed with a probability of 0.5. Note that by "diagnosis", we do not necessarily mean laboratory confirmed diagnosis of influenza - rather, we mean that an individual is symptomatic to a degree that triggers the interventions being modelled here. In the case of school closure, this means that a case in a pupil or teacher comes to the attention of the school or public health administration; in the case of antiviral interventions this means that the individual (or his/her) family seeks help from a source participating in the antiviral programme. The intervention measures considered are as follows.

### School closure scenarios

For each of the school closure strategies described below we assumed that 100% of individuals affected by school closure or isolation made none of their regular school hub contacts during the daytime cycle, but came into contact with other individuals present in their household, and made their usual community contacts (we assumed that no *additional *community contact occurred for these individuals - community contact was deemed to occur in all daytime cycles for active individuals, regardless of whether they were present at a hub or home, and this remained true during school closure). We also assumed that when a primary school aged child (age 5-12) was isolated in a household, one adult from the household stayed at home. We assume that these school closure *policies *are in place at the start of the local epidemic, but with the school case isolation and individual school closure strategies, actions to isolate groups or close only occur as cases appear in each school as described below.

#### School Case Isolation (SCI) with close contact group

We assumed that the diagnosed school case was isolated from the school along with a set of class members (approximate size of classes taken to be 30). The isolated individuals spent daytime cycles at home rather than at school for a given period ranging from 1 to 4 weeks, the intent being to reduce the potential spread of disease among the other school members. We further assumed that school case isolation (SCI) only applied to primary and high schools and not child care centres. This intervention was used in certain Australian states in the early stages of the A/H1N1 2009 pandemic [3].

#### Individual School Closure

In this intervention strategy we assumed that the whole primary school was closed if there was a case diagnosed. If there was an initial diagnosed case in a high school, the diagnosed case and class members were isolated from the school. If there was a further diagnosed case in another class then that case and class were also isolated. If there were more than two diagnosed cases in different classes in a high school then we assumed that the whole school would be closed. At the beginning of the pandemic this strategy was also followed in Australia, the United Kingdom and the USA [3,41,42].

#### All Schools Closure

This strategy assumed that all schools and child care centres in a community would be closed simultaneously after a certain number of cases (30, or 0.1% of the population) in the community had been diagnosed. This strategy was followed in Osaka and Hyogo in Japan [9].

Note that we assumed that school closure would be triggered at most twice for each school, on the grounds that repeated opening and closing of schools would be considered to be too disruptive, even for short periods of school closure. Our community model contained 22 schools.

### Antiviral drug scenarios

#### Treatment of Diagnosed Case (T)

We assumed that all diagnosed individuals would receive antiviral drug treatment at the time of their diagnosis. (24 hours after the appearance of symptoms) This treatment involved two doses taken daily for 5 days to reduce the infectiousness and infectious period of disease [19,20]. We assumed that treatment would reduce infectiousness of a symptomatic individual by 66% [38]. A further assumption regarding antiviral treatment is that that the duration of infectiousness of a diagnosed individual would be reduced by 1 day [19-22]. This intervention was initially used in certain Australian states at the beginning of the A/H1N1 2009 pandemic [3] and also used in USA, UK and Japan [41,42] [Yasuda2009].

#### Household Based Prophylaxis (T+H)

In this strategy we assumed that all diagnosed individuals would receive antiviral treatment. We further assumed that all household members of a diagnosed individual would be given antiviral drugs for prophylaxis, beginning at the same time as treatment of the diagnosed case, with each prophylaxis course consisting of one dose taken daily for 10 days [19-22]. We modelled the effect of prophylaxis on a susceptible individual as a reduction in susceptibility by 85% [38]. In addition, individuals who did become infected while taking prophylaxis had a 50% reduction in the chance of developing a symptomatic illness [3]. At the beginning of the pandemic this strategy was also followed by some states in Australia [2].

#### Extended Prophylaxis (T+H+E)

In this strategy the prophylactic use of antiviral drugs was extended to a wider group of contacts. Here prophylaxis courses were given to the class members (if the case is diagnosed in school) or to the mixing group (if the case is diagnosed in a workplace hub) of a diagnosed case in addition to their household members. This strategy was also followed in some states in Australia, in the UK and USA at the early stages of the pandemic [2,41,42].

Note that for all antiviral scenarios we assumed that a person would receive at most two prophylactic courses. For example, a person might receive a first course if a household member were diagnosed, and later might receive another course if a school classmate were diagnosed, but no further prophylactic course would occur if a third close contact were diagnosed. We also assumed that prophylaxis would not be administered once a person had experienced symptomatic infection. Treated individuals were not assumed to otherwise behave differently from other symptomatic individuals i.e. 50% of adults and 90% of children were assumed to withdraw to their household for the duration of their infection. All individuals taking prophylaxis courses were assumed to maintain their normal contacts during the daytime cycle.

In addition to determining the final and daily attack rates of the simulated epidemics, we also recorded the number of antiviral courses used. From this we also derived a measure of antiviral efficiency: the number of prevented cases per antiviral course. This was calculated as follows:

## Results

We initially simulated an unmitigated epidemic within the Albany community to determine the illness attack rate that would result if no interventions were in place. This was followed by an analysis of the effectiveness of the interventions, singly and in combination, via a series of simulation experiments which determined the reduction in attack rate which could be achieved by activation of the following: alternative school closure interventions without any antiviral-based interventions; alternative antiviral-based interventions without school closure interventions; and antiviral interventions in conjunction with school closure of different durations.

### Dynamics of disease spread under no-intervention scenario

Baseline no intervention simulations were conducted for reproduction numbers R_0 _ranging from 1.4 to 1.6, estimated to be the basic reproduction number for the A/H1N1 2009 pandemic [29,11]. The outcomes of simulated epidemics varied stochastically due to the random location of individuals who are seeded into the modelled community as infectious index cases and the probabilistic nature of infection transmission. Results for all simulated epidemics are averages of 40 runs, each with stochastic choices made with a different random-number sequence. This process is described in detail in [7]. As we assumed a continuous influx of infectious cases from outside the simulation boundary at a rate of one per day, we achieved a sustained epidemic for every simulation, following the approach adopted in [7]. Final attack rates ranged from 27% to 37% corresponding to R_0 _values of 1.4 to 1.6, respectively while peak daily incidence rates ranged from 82 to 159 cases per 10,000 people. The characteristics (means and standard deviations) of epidemics with an R_0 _of 1.5 are as follows: final infection rate (symptomatic and asymptomatic) of 43.9% (S.D. 1.09); final attack rate (symptomatic) of 32.5% (S.D. 0.77); peak ill population 6.6% (S.D. 0.46); peak daily illness case load (per 10,000) of 121 cases (S.D. 9); day of epidemic peak, day 45 (S.D. 4.74); serial interval 2.47 days (S.D. 0.01). The derived serial interval depends upon the latent and infectious durations and on the transmissibility of the virus. Our assumptions about these durations were based on seasonal influenza; when combined with a transmissibility calibrated to give an R_0 _of 1.5 (which we adopt in this study), the resulting serial interval of 2.47 days is also consistent with estimates of 1.3 - 2.71 days for the 2009 pandemic [29,9]. The characteristics of epidemics with R_0 _values of 1.4, 1.5 and 1.6 are listed in Table 2. Note that these characteristics apply to an epidemic within a community with the specific structure of our modelled community (that is, age distribution, distribution of household sizes and structures, number and size of school and workplace hubs and so forth).

**Table 2:** Simulated characteristics of baseline (no-intervention) epidemics for R_0 _values

	R_0_					
	1.4		1.5		1.6	
	
Characteristics	Mean	**S.D**.	Mean	**S.D**.	Mean	**S.D**.
Final Infection Rate (%)	36.3	1.32	43.9	1.09	50.4	1.02
Final Attack Rate (%)	26.9	0.95	32.5	0.77	37.2	0.74
Peak Symptomatic Population (%)	4.45	0.49	6.6	0.46	8.74	0.51
Peak Daily incidence Rate (per 10000)	82	8	121	9	159	10
Peak Attack Day	51	7.7	45	4.74	40	4.48
Serial Interval	2.49	0.01	2.47	0.01	2.45	0.01

Means and standard deviations (S.D.) are for 40 simulation runs, each with stochastic choices made with different random-number sequences.

### Impact of school closure based interventions

**Figure 2:**
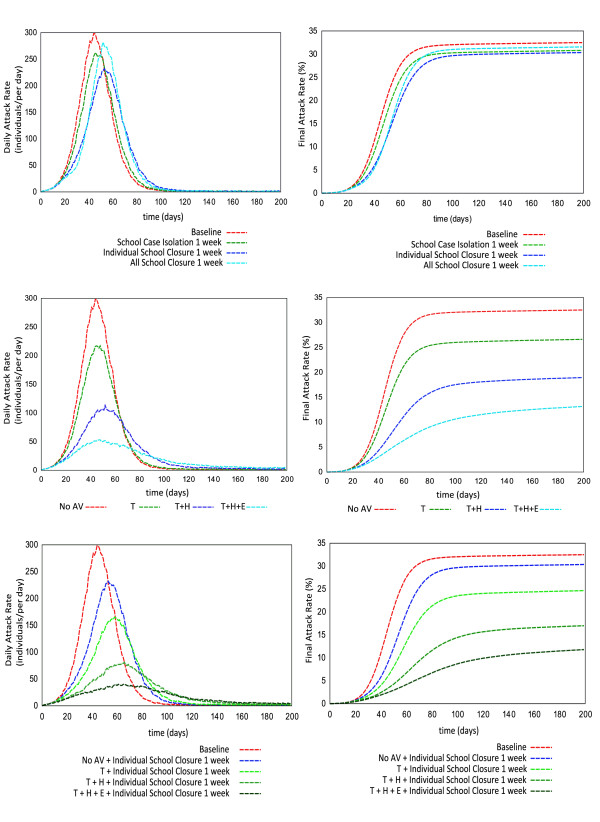
**Epidemic progression curve expressed as daily incidence rate and cumulative illness rate**. Top pair shows comparison among different school closure strategies with 1 week closing period. Central pair indicates the potential impact of antiviral drug usage during simulated pandemic period. Bottom pair illustrates the combined effectiveness of individual school closure for 1 week and antiviral strategies during pandemic. Results are illustrated in terms of daily incidence rate (symptomatic cases per day, left column) and cumulative symptomatic attack rate (percentage of population, right column).

We investigated the impact of school closure based interventions for an epidemic with an R_0 _of 1.5, the mid-point in the range of estimated basic reproductive numbers for A/H1N1 2009 influenza [29,11]. The results of different school closure interventions (Figure 2, daily incident rate (top left) and final attack rate (top right)) show that 1 week of school closure (for a maximum of two closures, that is a school may close, reopen and then close again) has minimal effect on reducing the epidemic size. A 2.5% cumulative attack rate reduction (from a baseline attack rate of 32.5% to an attack rate 30%) can be achieved by the *Individual School Closure *strategy, for example. When considering the daily incidence rate, there is also minimal benefit among the alternative school closure strategies of 1 week's duration (Figure 2, top left). However previous work suggests that school closure for longer durations may have a significant impact on reducing epidemic severities in terms of both the cumulative attack rate and the peak daily incidence rate [7].

### Impact of antiviral based interventions

Our simulations suggest that antiviral drug treatment for diagnosed symptomatic cases and its prophylactic use in close and extended contact groups can significantly reduce the size and severity of a local epidemic. For an influenza virus with a reproductive number of R_0 _= 1.5 and a diagnosis rate of 50% of all symptomatic individuals (a possible, realistic assumption) the antiviral treatment of diagnosed cases (with no prophylaxis to close contacts) reduced the overall illness attack rate by 6.5% from the unmitigated attack rate 32.5% to 26%. A further reduction in attack rate can be observed from simulation experiments if household prophylaxis and extended prophylaxis are in effect during the pandemic period. A reduction of 13.5% of symptomatic cases can be achieved (32.5% to 19%) using a household prophylaxis *(T+H) *strategy and a 19.5% reduction (32.5% to 13%) achieved if an extended prophylaxis *(T+H+E) *strategy is used (Figure 2, centre right). These antiviral-based pharmaceutical interventions also reduce the daily incidence rate (Figure 2, centre left, per 10,000). Without any concurrent school closure, a treatment-only *(T) *strategy results in a reduction of 33 symptomatic cases in the epidemic peak (from 120 to 87), a treatment and household prophylaxis strategy *(T+H) *gives a peak reduction of 74 symptomatic cases (from 120 to 46), while the treatment and extended prophylaxis strategy *(T+H+E) *gives a 96 case reduction in the epidemic peak (from 120 to 24), all per 10,000 individuals.

### Impact of antiviral based interventions combined with individual school closure

**Figure 3:**
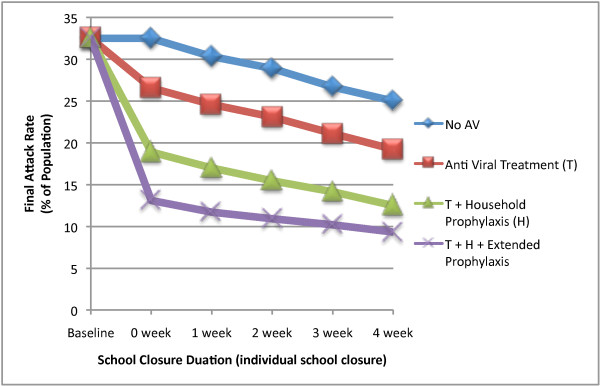
**Final attack rate of epidemics with concurrent school closure and antiviral based strategies**. Final attack rate (% of population) is presented for an epidemic with R_0 _= 1.5 and different antiviral based strategies in conjunction with individual school closure for variable school closure durations (0 week to 4 weeks).

We then examined the coupling of the three antiviral strategies with the *Individual School Closure *strategy for school closure periods of 1 to 4 weeks. For each of the antiviral strategies simulated, longer periods of school closure resulted in essentially linear reductions in cumulative attack rates; from the baseline unmitigated attack rate of 32.5% 4 weeks of school closure resulted in final attack rates of 25%, 19%, 12% and 9% for closure with no antiviral therapy, and combined with the *T*, *T+H *and *T+H+E *strategies respectively (see Figure 3).

**Figure 4:**
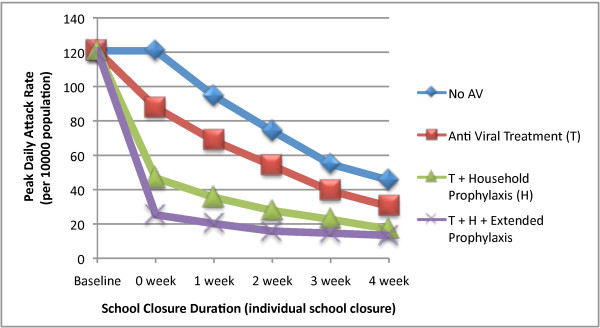
**Peak daily incidence rate of epidemic with concurrent school closure and antiviral based strategies**. The figure illustrates the impact of different durations of school closure coupled with antiviral based strategies on peak daily incidence rate during an epidemic. Peak daily incidence rate is expressed in symptomatic cases per 10000 population.

The combined strategies also gave significant reductions in daily case incidence rate. From the baseline unmitigated peak daily incidence rate of 120 (per 10,000) 4 weeks of school closure resulted in peak daily incidence rates of 45, 30, 18 and 14 with no antivirals, and combined with theT, T+H and T+H+E antiviral strategies respectively (see Figure 4). In the case of the treatment and treatment plus household prophylaxis strategies the addition of 4 weeks of school closure results in more than twice the reduction in peak daily incidence rate when compared to the use of antivirals without school closure.

### Impact on the antiviral stockpile

**Figure 5:**
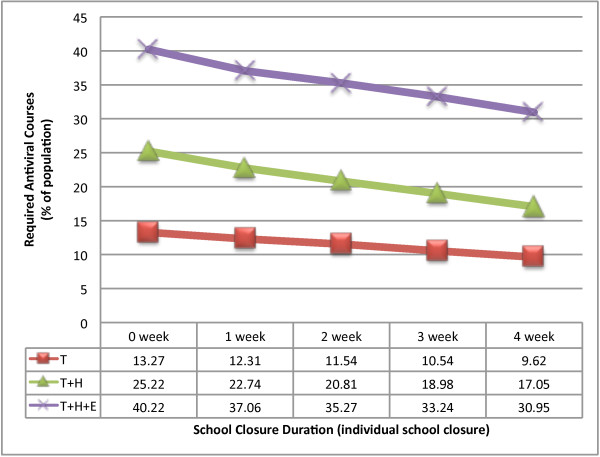
**Required antiviral courses (% of population) for an epidemic with R_0 _= 1.5**. The figure illustrates the consumption of antiviral drugs for different antiviral strategies combined with school closures during a pandemic.

Our results indicate that more antiviral courses would be consumed by the extended antiviral prophylaxis strategy (*T+H+E*) compared to the antiviral treatment (*T*) and household antiviral prophylaxis (*T+H*) strategies. An extra 14% (21% to 35%) of antiviral courses is required to achieve an additional 4% reduction (15% to 11%) in the final attack rate with the *T+H+E *strategy compared to the *T+H *strategy when both interventions are coupled with *Individual School Closure *of 2 weeks. The required ratio of antiviral courses to the population size is relatively higher using the extended antiviral prophylaxis strategy than the other antiviral based strategies (see Figure 5). We have presented the amount of antiviral courses as a % of the total population; therefore our results are scale free and are directly applicable to larger populations than that simulated. The number of prevented cases per antiviral course is 0.44 for the treatment strategy, 0.55 for treatment and household prophylaxis and 0.48 using the treatment and extended prophylaxis strategy, all assuming no school closure.

The summarized results giving Final Attack Rate (FAR), Peak Daily Incidence Rate (PDIR) and Required Antiviral Courses (RAV), expressed as a percentage of the population for each of the different intervention policies are listed in Table 3.

**Table 3:** Simulated outcomes for epidemics with different interventions

	R_0_								
	1.4			1.5			1.6		
	
Intervention Policies	*FAR*	*PDIR*	*RAV*	*FAR*	*PDIR*	*RAV*	*FAR*	*PDIR*	*RAV*
No intervention	27.9	82	0	32.5	121	0	37.2	159	0
ISC	23.5	53	0	29.3	74	0	34.1	100	0
T	21.1	54	10.4	26.6	84	13.3	32.2	120	16.0
T+H	12.6	24	18.4	18.7	41	25.2	23.4	62	30.3
T+H+E	9.0	15	30.6	13.0	22	40.2	19.9	39	45.8
T + ISC	16.9	32	8.5	23.8	53	11.5	27.9	77	14.1
T + H + ISC	10.1	14	14.2	14.8	23	20.8	20.8	41	26.9
T + H + E + ISC	7.1	10	25.0	10.9	17	35.3	15.5	30	44.8

Simulated *Final Attack Rate % (FAR)*, *Peak Daily Incidence Rate per 10,000 population (PDIR) *and *Required Anti Viral courses % (RAV) *for epidemics with different interventions. Interventions abbreviations: T - antiviral treatment, H - household antiviral prophylaxis, E - extended prophylaxis, ISC - individual school closure (2 weeks duration, maximum of 2 closures per school).

## Discussion

Using a detailed individual based model of an actual community we have evaluated interventions which were administrated during the A/H1N1 2009 pandemic in Australia and other countries. We have analysed the effectiveness of school closure and the use of neuraminidase inhibitor antiviral interventions as a means of reducing the number of infected individuals. Social distancing and antiviral drug interventions were the only public health measures available during the first 6 months of the pandemic, due to the initial lack of a suitable vaccine. These strategies, deemed as key strategies in the pandemic preparedness plans of the United States, United Kingdom, Australia and elsewhere [4,5,3], have been shown by previous modelling studies to play a prominent role in the early containment of a future H5N1 pandemic [20,22-24]. In the modelling study presented here we have examined the potential benefit of the *sustained *use of a range of antiviral drug and school closure strategies, suggesting strategies which may be more effective than those actually adopted.

Our results give guidance as to which mitigating, control and containment strategies may perform better than those used in the early phases of the 2009 pandemic. While the 2009/2010 influenza pandemic has been classified as *mild *by the World Health Organisation and the Centre for Disease Control and Prevention [43,41] these results will have relevance to future influenza pandemics which may be significantly more pathogenic, with higher case fatality rates than those seen in 2009. The results are thus of relevance to public health authorities as they digest the lessons learned from the 2009 outbreak and their responses to it.

At the early stage of the 2009 pandemic strategies such as school closures and antiviral drug application for treatment and prophylaxis were used in many countries with the intent of containing disease spread. These strategies appeared to have had only limited success, with the notable exception of Japan where early, large scale closure of schools suppressed an outbreak that was spreading rapidly amongst school-aged children [43]. Here the application of 1-2 weeks of school closure contained an outbreak stemming from a small number of early imported cases, and consequentially the progress of the pandemic was delayed by approximately 6 weeks [34].

In many countries there was an apparent hesitancy to use antiviral drugs aggressively and extensively, even at the early stages of the pandemic. This hesitancy may have been due to either the fear of running out of an antiviral stockpile while emergence of human transmissible H5N1 is still a real risk, or may have been due to worries regarding emergence of antiviral-resistant strains of the A/H1N1 2009 influenza virus [43,44]. The approach taken by many countries in 2009 generally involved use of antiviral drugs for treatment and (limited) prophylaxis coupled with contact tracing during the initial stages, quickly changing to treatment only and then to partial treatment (i.e. only some diagnosed cases receiving antiviral treatment). In the initial stages the use of antivirals was often coupled with 1 week of school closure or class isolation at home [45]. We have modelled these strategies together with more rigorous strategies to determine (1) whether the strategies used would have been effective from a contain and control perspective and (2) whether more effective strategies, in terms of reduction in attack rate, can be determined. The better strategies suggested by our results involve a more aggressive and sustained use of antiviral drugs (for both treatment and prophylaxis) and longer school closures than that which occurred.

The results indicate that a treatment and prophylaxis strategy generates a greater reduction in the number of cases when compared to treatment alone, with the extended strategy working best. However such a strategy requires a significantly larger antiviral drug stockpile than treatment-only or treatment and household-only prophylaxis. Given the financial limits faced by many countries in creating and maintaining antiviral stockpiles our results suggest that a *sustained *treatment and household prophylaxis strategy appears optimal, in terms of the ratio of illnesses avoided to antiviral courses required. This strategy is feasible if a stockpile of 252,000 antiviral courses is available per 1 million of a population, which may be determined from our results on stockpile size. If a larger stockpile is available, or it can be rapidly replenished, then a sustained treatment plus extended prophylaxis regimen is recommended. In this case, our results estimate that a stockpile of 402,000 courses per 1 million of a population would be required. If no additional school closure is to be utilized 132,000 courses are required for treatment-only per 1 million of the population.

However if school closure is also activated, such as closure of individual schools for 2 weeks, then the required stockpile of antiviral drugs reduces to 115,000 courses for a treatment-only antiviral strategy, 208,000 for treatment and household prophylaxis and 352,000 for treatment and extended prophylaxis, again per 1 million population.

These results are applicable for a pandemic with a basic reproduction number R_0 _of 1.5 and a plausible diagnosis rate of 50% of symptomatic individuals, with diagnosis occurring 24 hours post symptom appearance. As diagnosis is assumed to be necessary for antiviral treatment (and contact prophylaxis) to occur, rapid diagnosis may only be possible if diagnosis is based on influenza-like illness (ILI) symptoms rather than serological testing.

Our simulations suggest that treatment with household prophylaxis may be the better strategy. When coupled with 2 weeks of school closure, the treatment plus household prophylaxis strategy has the greatest number of prevented cases per course ratio (0.85 cases/antiviral course), compared to treatment only (0.75 cases/antiviral course) and the treatment plus extended prophylaxis strategy (0.61 cases/antiviral course). Using this measure to determine optimal use of an antiviral stockpile, that is the prevented cases to antiviral courses required ratio, the treatment plus household prophylaxis strategy may be considered optimal. In addition, it may reduce the chance of antiviral resistance development given the lesser amount of prophylaxis occurring compared to the extended strategy. While little antiviral drug resistance to A/H1N1 was detected during the 2009 pandemic, the level of antiviral used which this study indications would be required for sustained prophylaxis strategies may be significantly increase the chance of antiviral resistant strains emerging.

With regard to the effectiveness of school closure strategies our results suggest that the closure of individual schools is the preferred strategy, compared to when all schools close simultaneously or one of only removing and isolating diagnosed cases and their contacts. That the individual school strategy out-performs the all-school closure strategy is due to its adaptive nature; individual school closure is better tuned to where (the individual school) and when cases within individual schools arise. Closing all schools together is less than optimal as some of the schools closed may well have no infectious cases present. For the three school closure strategies examined we find that closure of one week has limited impact and closure of two or more weeks is increasingly more effective, in a linear manner.

In the absence of intervention our model assumes an age-specific attack rate that is higher in children and young adults (see Methods section), which is consistent with A/H1N1 2009 influenza [37]. Previous modelling studies, such as prior work by the authors with the same simulation model used here (see [7] supplementary info), and others [17], have found that, as might be expected, school closure is less effective in the case of uniform age-specific attack rates.

### Related research

We have identified 12 previous simulation studies that deal with the use of antiviral interventions for pandemic influenza mitigation [19,26,22,24,46,25,14-27,47,37]. Five of these can be meaningfully compared to our study, being both methodologically similar (individual-based models) and examining similar interventions at similar reproduction numbers [20,22,26,25,47]. Four of these found results broadly consistent with our results [20,26,25,47]; these used baseline (unmitigated) epidemics with final attack rates in the range 30%-34.5% and found that antiviral treatment plus prophylaxis to households resulted in final attack rate reductions of 41%-54%, requiring antiviral stockpile sizes of from 410,000 to 580,000 per million population. Our comparable result finds a similar attack rate reduction (40%) but is more optimistic in terms of stockpile requirement (250,000). This may arise as a consequence of our assumption of a higher prophylactic efficiency (85%, compared to 30% for these studies), which is based on a more recent analysis of antiviral effectiveness studies [38,40]).

The work of Germann et al [22] appears to be an outlier, finding the treatment plus household prophylaxis strategy overwhelmingly more effective, essentially completely preventing an epidemic (final attack rate of 0.06% from 33% baseline) while using only a 10,000 per million stockpile. McCaw and McVernon [14], using a differential equation-type of model, find that a treatment and extended prophylaxis strategy could very effectively postpone an epidemic. They assume a lower R_0 _than the 1.5 used here (for the same final attack rate) and note that their model is very sensitive to seeding (or intervention delay) assumptions. Also, the use of contact tracing of 20%-40% of contacts (required for the long epidemic postponements) for a uniform mixing model cannot be directly compared to an individual-based model; tracing from 4 to 8 out of 20 assumed contacts spread randomly through the population may represent a harder task than tracing 15 or so household and hub members directly in contact with a diagnosed case. The study reported in [27], while comprehensive, is not comparable with our study for the following reasons: the lowest R_0 _considered was 1.7, the diagnosis ratio assumed was 60% or 80%, all scenarios included additional social distancing (in the workplace and wider community) which was not widespread in 2009. Furthermore that study did not consider the T or T+H+E strategies and did not report on antiviral stockpile requirements.

Unlike antiviral medication, school closure has historically been used as an intervention measure in influenza pandemics, usually as part of a "social distancing" policy. Evidence from reviews of past studies [48,28] shows that some benefits can be obtained from the closure of schools during seasonal and pandemic influenza. However there is still substantial debate about if, when and how school closure should be implemented during a pandemic. Previous simulation studies have been performed to address the issue of the potential effectiveness of school closure, with varying results [20,22,23,49]. A comparative study of different simulated school closure interventions [7] concluded that effectiveness depends on several factors about which little is known, such as the relative proportion of infections that occur in the school setting, the contact behaviour of pupils when school closure is in effect, and the timing and duration of school closure. In addition to the uncertain effectiveness of school closure, it is also unclear which type of school closure policy might be economically and socially acceptable in a community during a pandemic. This motivated us to improve upon previous work by closely modelling several school closure strategies which actually took place at the early stages of the 2009 influenza pandemic and we focussed on three particular school closure/class isolation strategies. As information about the contact behaviour of pupils during school closure during the 2009 pandemic is only now appearing [45], we made the assumption that pupils would make household and community contacts during school closure, but no additional compensating contacts. Since the school closure strategies we simulated actually occurred, we can be sure that they represent practical and generally acceptable measures. Although the interventions which were implemented were generally of short duration, we speculate that if the 2009 pandemic had exhibited a higher case fatality ratio, longer durations of school closure would have been tolerated, or even demanded.

In the study reported here we have not modelled other non-pharmaceutical interventions such as household quarantine, workplace closure or public gathering bans, interventions that were much less in evidence during the 2009 pandemic. This is not because we believe that these interventions are ineffective or unimportant; on the contrary, combinations of rigorous social distancing interventions may be the only way to arrest the spread of a highly transmissible, highly pathogenic influenza strain until vaccines are available, as determined and discussed in [7,8].

## Conclusions

The results suggest that antiviral drugs may be utilized in a more effective manner, and have significantly more impact for containing an influenza pandemic, than that which occurred during the 2009 pandemic. Specifically, the *sustained *use of antiviral drugs for treatment and prophylaxis appears to be a better strategy than that adopted in many countries, which restricted its use to treatment-only at the early stages of the pandemic. These results furthermore suggest that creating an antiviral drug stockpile of a size which allows a treatment plus household antiviral strategy is optimal in terms of cases averted per antiviral course. Simulation of a range of school closure strategies and durations suggests that isolating diagnosed individuals and contacts is less effective than closing whole schools, but that they need to be closed for periods of at least two weeks for there to be a significant benefit. Closure for periods beyond three weeks is even more effective but is probably only feasible from a societal perspective if the pandemic has a high case fatality ratio. The coupling of school closure with each of the three antiviral strategies improves their effectiveness in reducing both the daily incidence rate and the overall attack rate. This indicates the value of using school closure strategies, which were used in a limited manner during the 2009 pandemic.

## Competing interests

The authors declare that they have no competing interests.

## Authors' contributions

NH, JK and GM were responsible for the conception and design of the simulation experiments. NH and JK were responsible for software development. NH conducted simulation experiments. All authors were involved in the analysis of simulation results and writing the manuscript; all authors read and approved the final manuscript.

## Pre-publication history

The pre-publication history for this paper can be accessed here:

http://www.biomedcentral.com/1471-2458/10/168/prepub
